# In Vivo and In Silico Study of the Antinociceptive and Toxicological Effect of the Extracts of *Petiveria alliacea* L. Leaves

**DOI:** 10.3390/ph15080943

**Published:** 2022-07-29

**Authors:** Kelly del Carmen Cruz-Salomón, Rosa Isela Cruz-Rodríguez, Josué Vidal Espinosa-Juárez, Abumalé Cruz-Salomón, Alfredo Briones-Aranda, Nancy Ruiz-Lau, Víctor Manuel Ruíz-Valdiviezo

**Affiliations:** 1 Tecnológico Nacional de México, Campus Tuxtla Gutiérrez, Tuxtla Gutierrez 29050, Mexico; i.bq.kelly@gmail.com (K.d.C.C.-S.); nruizla@conacyt.mx (N.R.-L.); bioqvic@hotmail.com (V.M.R.-V.); 2 Escuela de Ciencias Químicas, Universidad Autónoma de Chiapas, Ocozocoautla de Espinosa 29140, Mexico; dr.abumale@gmail.com; 3 Facultad de Medicina Humana, Universidad Autónoma de Chiapas, Tuxtla Gutierrez 29090, Mexico; alfred725@hotmail.com

**Keywords:** *P. alliacea*, pain, antinociceptive, phytochemical composition, plant extract

## Abstract

*Petiveria alliacea* L. is an herb used in traditional medicine in Mexico and its roots have been studied to treat pain. However, until now, the antinociceptive properties of the leaves have not been investigated, being the main section used empirically for the treatment of diseases. For this reason, this study aimed to evaluate the antinociceptive and toxoicological activity of various extracts (aqueous, hexanic, and methanolic) from *P. alliacea* L. leaves in NIH mice and to perform an in silico analysis of the phytochemical compounds. Firstly, the antinociceptive effect was analyzed using the formalin model and the different doses of each of the extracts that were administered orally to obtain the dose–response curves. In addition, acute toxicity was determined by the up and down method and serum biochemical analysis. Later, the phytochemical study of extracts was carried out by thin layer chromatography (TLC) and visible light spectroscopy, and the volatile chemical components were analyzed by gas chromatography-mass spectrometry (GC/MS). Moreover, the most abundant compounds identified in the phytochemical study were analyzed in silico to predict their biological activity (PASSonline) and toxicology (OSIRIS Property Explorer). As a result, it was known that all extracts at doses from 10 to 316 mg/kg significantly reduced the pain response in both phases of the formalin model, with values of 50–60% for the inflammatory response. The toxicological studies (DL50) exhibited that all extracts did not cause any mortality up to the 2000 mg/kg dose level. This was corroborated by the values in the normal range of the biochemical parameters in the serum. Finally, the phytochemical screening of the presence of phenolic structures (coumarins, flavonoids) and terpenes (saponins and terpenes) was verified, and the highest content was of a lipid nature, 1.65 ± 0.54 meq diosgenin/mL in the methanolic extract. A total of 54 components were identified, 11 were the most abundant, and only four (Eicosane, Methyl oleate, 4-bis(1-phenylethyl) phenol, and Ethyl linolenate) of them showed a probability towards active antinociceptive activity in silico greater than 0.5. These results showed that the *P. alliacea* L. leaf extract possesses molecules with antinociceptive activity.

## 1. Introduction

Pain is one of the main causes of medical care because it is associated with different diseases. The International Association for the Study of Pain (IASP) defines pain as an unpleasant sensory and emotional experience associated with, or like that associated with, actual or potential tissue damage [1]. Some species of plants have been used in traditional medicine for the treatment of pain [2].

In Mexico, the species *Petiveria alliacea* L., known by the common names: wild warbler, chicken grass, japachumi, skunk branch, skunk, wild skunk, and fox grass, have been used empirically to relieve muscle pain and mouth pain, among others. It is a plant native to the southern United States and Mexico [3], which generally grows in humid, somewhat shady, and riverside places [3,4]. *P. alliacea* L. is a perennial herbaceous plant that reaches up to 1 m in height, its leaves are alternate, oblong to elliptic, acute to acuminate at the apex, and 6–19 cm long and generally glabrous. The flowers are small, arranged in axillary or terminal spikes, 15–40 cm long, graceful, small, hermaphrodite, with four petals, white, greenish-white, or light pink; its fruit is about 8 mm long, cuneate, striated, with six terminal bristles; solitary, linear seed [4,5].

Some ethnopharmacological studies have been reported based on surveys and empirical knowledge, presenting evidence of the anti-inflammatory and antinociceptive activity of this plant [6,7,8]. In addition, a pre-clinical investigation with *P. alliacea* L. root extracts showed the potential antinociceptive effects using various models in mice, such as abdominal constriction induced by acetic acid, hot plate, or formalin tests [9]. In addition, the lyophilized ethanolic extract from the root of this same plant, evaluated by the carrageenan model and the Swingle method in rats, was related to anti-inflammatory and antinociceptive effects [10]. However, the use of the root sacrifices the plant, limiting its continuous use. On the other hand, some studies have examined the metabolites with different biological properties of *P. alliacea* L. [11,12], but there are no studies demonstrating possible antinociceptive activity in leaves. In addition, there are several previous studies on the toxicity of aqueous extracts from the leaves of this plant [13,14]. However, it is necessary to broaden the polarity ranges, in order to have a greater diversity of metabolites to be evaluated. Therefore, this research aimed to evaluate the antinociceptive and toxicological activity of various extracts (aqueous, hexanic, and methanolic) from *P. alliacea* L. leaves in NIH mice and to perform an in-silico analysis of the phytochemical compounds.

## 2. Results

### 2.1. Extract Yield

Yield percentages show the amount of extract obtained using different solvents per 100 g of plant material. The highest percentage of yield was obtained with the aqueous extract (30.1%), the second percentage with the methanolic extract (9.5%), while the lowest yield was obtained with the hexanic extract (2.7%).

### 2.2. Evaluation of Antinociceptive Activity

**Figure 1:**
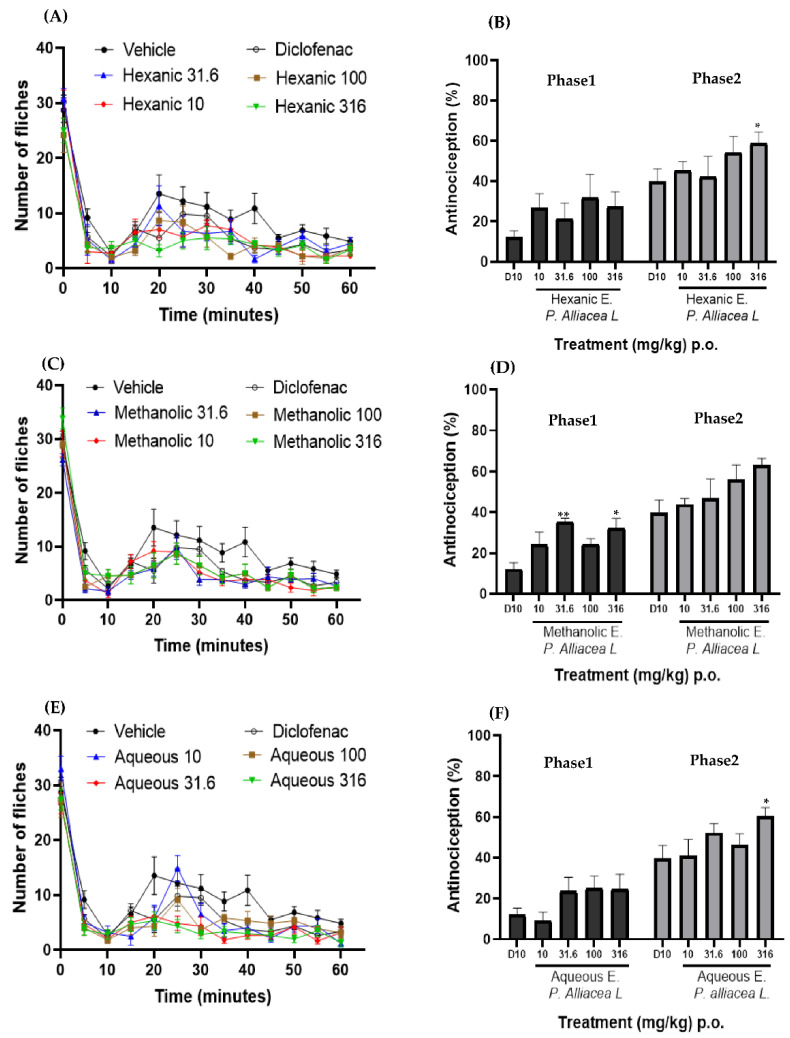
Subfigures show the time courses (**A**) hexanic extract, (**C**) methanolic extract and (**E**) aqueous extract, each point represents the mean ± S.E.M. *n* = 6/group. Subfigures represent the dose-response graphs expressed as area under the curve of the time course corresponding to (**B**) hexanic extract, (**D**) methanolic extract and (**F**) aqueous extract of the *P. alliacea* L. leaves. Bars are the mean ± S.E.M., *n* = 6/group. * *p* < 0.05; ** *p* < 0.01 vs. D10 (diclofenac 10 mg/kg) as determined by one-way ANOVA followed by Tukey Test.

The antinociceptive activity was evaluated using the formalin model, where the time courses of each of the doses of the different extracts, the controlled drug and the vehicle, were obtained. With this model, the two phases of pain can be seen (Figure 1A,C,E), the first phase is neurogenic, corresponding to minutes zero to ten. After this phase, there is a brief period of stillness, followed by the second phase that is inflammatory, which develops from 15 to 60 min.

Once the time course was constructed, the area under the curve of both phases were obtained using the trapezoid method. Subsequently, with the help of these areas, the percentage of antinociception observed in Figure 1B,D,F was calculated in their respective doses of the different extracts and the control drug. In addition, a one-way ANOVA was performed and compared with diclofenac (D10), where an asterisk (*) represents a statistical difference with *p* < 0.05 and two asterisks (**) with *p* < 0.01, and where there are no asterisks it means that there are no statistical differences with D10.

The time course of the different doses of the hexanic extract is shown in Figure 1A, where it is observed that none of the doses evaluated is above the course of the vehicle, but they have a similar behavior because there is no pain inhibition. From the initial course, the dose–response graph was made (Figure 1B), hence observing that during phase one, there is no significant statistical difference between the different doses of the hexanic extract and the control drug. This extract presented an antinociceptive activity between 25% and 31%. In phase two, it was observed that the dose of 316 mg/kg presents a statistically significant difference (*p* < 0.05) with concern to D10. On the other hand, this phase achieved an activity of 42–60%.

Figure 1C shows the time course of the different concentrations of the methanolic extract where it is observed that the course of the vehicle is greater than the doses evaluated because there is no inhibition of pain. On the other hand, the control drug and the different doses have a similar number of shocks while the evaluation time elapses. Therefore, similar behavior is observed between them and neither exceeds the vehicle. In the dose–response graph of the methanolic extract (Figure 1D), it can be seen that in phase one, the doses of 31.6 and 316 mg/kg present statistically significant differences (*p* < 0.01 and *p* < 0.05, respectively) with the drug control. However, in phase two of the evaluation, only the dose of 316 mg/kg presents a statistical difference with *p* < 0.05.

Finally, the aqueous extract was evaluated, obtaining the time course shown in Figure 1E. Here, it is observed that from the application (time 0) until minute 25, the dose of 10 mg/kg exceeds the number of shakes of the vehicle, but statistically, it is not significant, so at these points, there is no analgesic effect. Once the time course was obtained, the dose–response graph was obtained (Figure 1F), with the result in phase one that the treatments did not present a significant statistical difference with respect to the control drug, with this extract reaching an approximate inhibition of 25%. Furthermore, in phase two, we can observe that the dose of 316 mg/kg has a statistically significant difference (*p* < 0.05) with concern to D10, as well as a tendency to increase antinociceptive activity, reaching 60%.

### 2.3. Toxicity Test

To determine the toxicity of the different extracts of the leaves of *P. alliacea* L., the “up and down” method was used once the treatment (2000 mg/kg) was administered, and a physical and behavioral analysis was carried out, in the acute evaluation. During the first 3 h and up to day 15 post-administration days, no rodents showed symptoms of aggressiveness, tremors, paralysis or skin lesions. Additionally, it was observed that there was no mortality in the groups with the administration of the different extracts, so it was determined that the median lethal dose (LD_50_) is greater than 2000 mg/kg.

The mice were sacrificed 15 days after the administration of the single dose of 2000 mg/kg of each of the extracts, and serum was obtained for biochemical evaluations (Table 1). Regarding glucose levels, the values are within the normal range and there is no statistically significant difference between the crude extract treatments and the control. On the other hand, total protein was used to evaluate the liver status, finding that the use of the methanolic extract decreased the values but within the normal range, which is corroborated with the data obtained from albumin, since there is no significant statistically difference between the different groups. The groups treated with the methanolic and hexanic extracts showed low amylase content without leaving the normal range, allowing us to consider that the functioning of the pancreas is correct, because it is adequately synthesizing this enzyme.

### 2.4. Identification of Metabolites in P. alliacea L. Leaves

The phytochemical composition of *P. alliacea* L. leaves, from methanolic, hexanic, and aqueous extracts, were analyzed, identifying different families of metabolites, such as flavonoids, saponins, terpenes, coumarins, anthrones, anthraquinones, and alkaloids.

The concentrations of the different metabolites present in each of the respective extracts *P. alliacea* L. leaves are shown in Table 2. It is observed that there is a significant statistical difference between the extracts and the highest concentrations for most of the compounds extracted from the leaves of *P. alliacea* L. are obtained with the methanolic extract, except for total phenols. On the contrary, in the aqueous extract a higher concentration of saponins, total phenols, and terpenes could be observed, while in the hexanic extract a higher concentration of terpenes was observed, possibly due to the chemical nature of these molecules that have a greater affinity for apolar substances.

#### 2.4.1. Identification of Components by GC-MS

**Figure 2:**
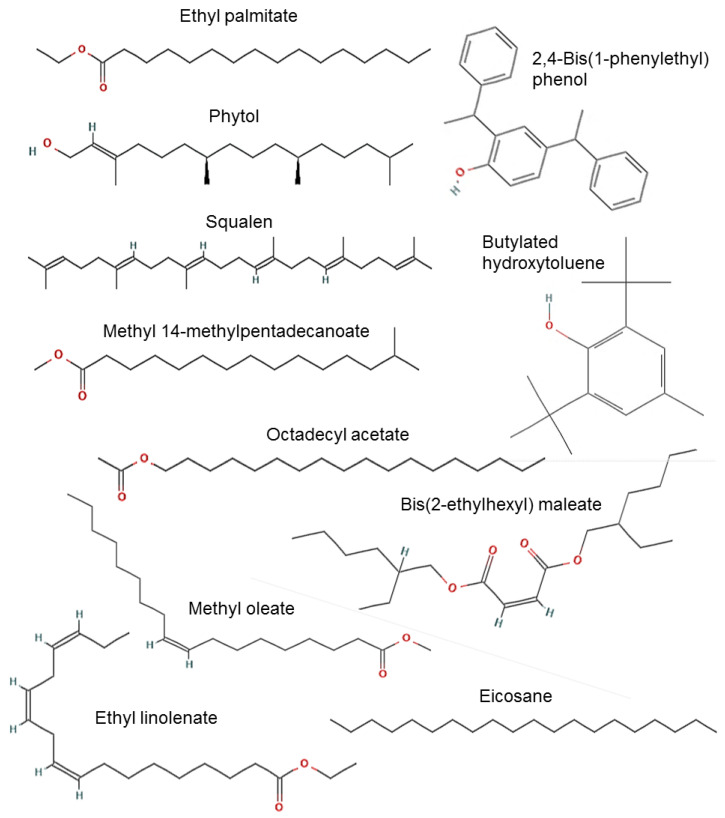
Structure of the most abundant molecules in the *P. alliacea* L. leaves.

GC-MS analysis allowed the detection and identification of 54 compounds in total, but 11 of them were the most abundant (Table 3, Figure 2). In the methanolic extract, 16 compounds were identified such as: butylated hydroxytoluene; 4-cyanocinnoline; 1-(3,5-dimethyl-1-adamantanoyl) semicarbazide; imidazole 2-amino-5-[(2-carboxy)vinyl]; asteromycin; 4-fluorohistamine; 4-methoxyamphetamine; 2-piperidinone-1-methyl; 3,3′-iminobispropylamine; 1-(5-bicyclo(2.2.1)heptyl)ethylamine; methypent-4-enylamine; 1*h*-indole-3-ethanamine 6-fluoro-beta-methyl.

For the hexanic extract, 16 compounds were identified as: 1-methyl-2-phenoxyethylamine; pentadecanoic acid-14-methyl methyl ester; 2-(2-carboxyvinil) pyridine, trans; hexadecanoic acid 14-methyl methyl ester; octadecanoic acid methyl ester; 1-adamantyl(phenyl)methanone thiosemicarbazone; tetratetracontane; benzene-1-chloro-4-(2-phenylethenyl); 1,2-benzenedicarboxylic acid mono(2-ethylhexyl) ester; tetrasiloxane decamethyl; cyclotrisiloxane hexamethyl; 5-acetamido-4,7-dioxo-4,7-dihydrobenzofurazan. 

Finally, 22 compounds were identified in the aqueous extract among which are: 2-methoxy-4-vinylphenol; 1-propanone 1-(1-adamantyl)-3-dimethylamino; phenol 2-(1-phenylethyl); acetamide 2-amino; 1,2-benzenedicarboxylic acid butyl octyl ester; 5-azauracil; 3-pyrrolidinecarboxylic acid 1-methyl-5-oxo; pentanamide *n*-decyl-*n*-methyl; 3-azahexan-1-ol 6-cyclohexyl; dodecane 1-fluoro; 2-butenedioic acid [E] bis(2-ethylhexyl) ester; 7-oxabicyclo(4.1.0)heptane 1,5-dimethyl; nonahexacontanoic acid; aluminun tripropyl; benzaldehyde 2-nitro diaminomethylidenhydrazone; 1*h*-indole 5-methyl-2-phenyl; cyclotrisiloxane hexamethyl.

#### 2.4.2. In Silico Analysis of the Most Abundant Compounds

The results for the analysis of the biological effects of the components found in the extracts of *P. alliacea* L. were obtained from PASSonline (Table 4). It was observed that all the compounds present, to a greater or lesser extent, a probability of acting as antinociceptive or anti-inflammatory, although a greater probability of anti-inflammatory activity can be noted, even being observed in some of them, such as Ethyl linolenate and Butylated hydroxytoluene, with Pa (probability to be active) greater than 0.80.

Derived from the previous analysis, an in silico toxicological analysis was carried out with the OSIRIS Property Explorer program regarding the different compounds found in the extracts, since this allows chemical structures to be drawn and various relevant properties of the compound to be calculated on the fly if the structure is valid. The prediction results are scored and color-coded, as shown in Table 5. Properties with a high risk of undesirable effects, such as mutagenicity, irritability, etc., are shown in red (high risk). Meanwhile, a green color (zero risk) indicates a behavior consistent with the compound. In addition, we can see that two compounds present a high risk of toxicity, such as butylated hydroxytoluene and bis (2-ethylhexyl) maleate.

## 3. Discussion

For the in vivo evaluation of the antinociceptive activity, the formalin model was used, which has two phases. The first called neurogenic is caused by the activation of the primary afferent fibers by the action of formalin. After this phase, a short period of quiescence, followed by the second phase related to the release of inflammatory mediators [15,16]. 

In the results, an attenuation of the nociceptive behavior was observed with the administration of the different antinociceptive activities of *P. alliacea* L., using the root [9,10]. However, this is the first evidence of the evaluation of the extracts used in the leaves of this plant. The range of percentages of antinociception found in this study for each phase of the evaluated method presents a behavior like that reported by Lopéz-Canúl, [17] who evaluated the *Verbesina persicifolia* leaves. During the neurogenic phase, the values are lower than those obtained in the anti-inflammatory phase. 

Analyzing the antinociceptive effects, some differences have been described with previous studies that have evaluated plant extracts in the formalin model. For example, in phase one, in this work, greater inhibition of pain was found compared to that reported by Xu et al. [18] in the evaluation of *Flos populi* flowers and by Díaz-Castillo [19], who analyzed the antinociceptive effects of *Macrolobium pittieri* leaves. However, the study results presented a lower percentage of inhibition compared to that reported by Hajhashemi et al. [20] who examined *Rosa damascena* petals. Meanwhile, in phase two, the study results were less than the effects of the aqueous extract of flowers of *Flos populi* [18]. These differences could be based on the variation of the bioactive metabolites present in each of the plant species. However, in phase two, the extracts from the *P. alliacea* L. leaves presented a higher percentage of pain inhibition compared to reported by Guerra et al. [21] in the evaluation of *P. lichnidiflora* leaves and by Díaz-Castillo [19], and a lower percentage compared to Xu et al. [18] and Hajhashemi et al. [20]. This may be because Hajhashemi et al. [20] used a dose of 1000 mg/kg, higher than that of this investigation. Moreover, the flowers of *Flos populi* could have more bioactive compounds that act in this phase, thus reducing the pain. 

The toxicological study, where no lethality was found with a high dose, is consistent with the lack of significant alterations in the levels of the biochemical indicators studied after administration with most of the extracts. This can consequently be interpreted as a plant that presents certain security for its use and it can be said that the average lethal dose of the different extracts of the *P. alliacea* L. leaves is above the dose evaluated. This result of the median lethal dose coincides with the work of García-Pérez et al. [13], where no mortality was observed with the dose of 2000 mg/kg. However, in this investigation, only the fractions of an ethanolic extract were evaluated, using the stem and leaves, while our study evaluated different extracts, allowing the extraction of a wide range of metabolites depending on their polarity, and thus reinforcing the evidence of safety in the use of the plant. 

The correlation with biochemical parameters in the serum of NIH mice was used to identify alterations that could be considered severe. An increase in glucose, consistent with some studies performed [14,22] but inconsistent with the Garcia-Perez et al. [13] investigation, was observed as a similar glucose concentration was maintained in all experiments. On the other hand, a decrease in triglycerides was observed in mice, which has not been studied, leaving open the possibility of future research related to this plant. Finally, a decrease in uric acid outside the normal range was observed. Despite this, it did not directly affect the LD_50_, probably because the concentration of some substances present in the extracts is not sufficient to generate mortality. This result agrees with Oyeleke et al. [23] and Muhammad et al. [24], who evaluated an aqueous extract of the root and leaves of this plant in pullet chicks.

In general, the result of the phytochemical analysis of *P. alliacea* L., is consistent with that previously reported in various studies [25,26,27]. However, it is important to highlight the novel presence of terpenes, anthrones, anthraquinones and coumarins, metabolites that were found in this study and that could represent new elements of study for future applied research. The differences between these new elements found compared to other studies could be based on multiple factors, such as collection time, environment, types of solvent and extraction method; factors widely discussed in previous reports [28].

Regarding the chromatographic analysis, the presence of 11 compounds with greater abundance in the extracts was evidenced, of which ethyl palmitate [29], phytol [30], ethyl linolenate [31], squalene [31], and methyl oleate [32] have been reported in the literature with antinociceptive and anti-inflammatory activity. On the other hand, there is no evidence of antinociceptive or anti-inflammatory activity of methyl 14-methylpentadecanoate, 2,4-bis(1-phenylethyl) phenol, butylated hydroxytoluene, eicosane, bis(2-ethylhexyl) maleate, and octadecyl acetate. However, various investigations [33,34] report the antioxidant activity of these compounds, which indirectly influences the reduction of pain, since it has been shown that in painful conditions there is an increase in free radicals, which promotes a higher concentration of intracellular calcium and a decrease in glutamate reuptake, causing an increase in the activation of the NMDA receptor, promoting sensitization at the central level [35,36]. So, a decrease in free radicals would favor the antinociceptive effects, coupled with the fact that in silico analysis, which makes a prediction based on structure–activity relationships, showed that the compounds have a probability of acting as antinociceptive molecules, thus opening up an area of opportunity to evaluate these bioactive compounds in isolation to confirm this pharmacological effect.

Finally, the in silico analyses were carried out through PASSonline, a platform that makes predictions of the biological activities of molecules through the structure–activity relationship, with an accuracy greater than 95% [37]. Molecules with a high probability of generating analgesic and anti-inflammatory effects are present in all the extracts. These findings correlate with some activities reported for the identified molecules, since it has been shown that they can act as antioxidants, inhibitors of lipoxygenase, and inhibitors of 5alpha-reductase [38,39,40], via mechanisms that are associated with pain reduction [39,41,42]. 

Adopting the toxicity alarm descriptors through in silico tests using the Osiris property program, two molecules with signs of risk were detected, butylated hydroxytoluene and bis (2-ethylhexyl) maleate. It has been reported that these molecules can cause cough, sore throat, reddening of the skin and eyes, abdominal pain, confusion, vertigo, nausea, vomiting, and prolonged or repeated exposure, which causes dermatitis. These can affect the liver, be carcinogenic, as well as hazardous to pregnancy [43,44]. However, since no mortality was found with a high dose of each of the extracts, it is possible that their concentration was not sufficient to cause these alterations in the conditions evaluated, in addition to the fact that the effects evaluated were with acute dosages.

## 4. Materials and Methods

### 4.1. Collection of Plant Material

*P. alliacea* L. leaves were collected from several randomly distributed individuals in the same phenological stage (autumn) from the municipality of Ocozocoautla de Espinosa (N 16°45′07.4″ and W 93°22′22.6″) located in the state of Chiapas, Mexico. The botanical identification took place at the herbarium of the Botanical Garden “Dr. Faustino Miranda” with the registration number 54016. 

### 4.2. Obtaining the Extract

*P. alliacea* L. leaves were dried at 45 °C in a Cole-Parmer StableTemp oven and ground manually. The mixtures with organic solvents (methanol and hexane) and the aqueous were prepared in a 1:10 ratio and were subjected to sonication for 2 h in a Cole-Parmer 08855-00 sonicator (Illinois, USA). Then, each of the mixtures was filtered by vacuum with a Welch Vacuum Gem 8890A-70 pump with Bluffton 1603007402 motor and centrifuged at 5000 rpm for 15 min in a Solbat J-600 centrifuge (Puebla, Mexico). Only the extracts with the organic solvents were concentrated under reduced pressure in a Büchi R-210 rotary evaporator (Flawil, Switzerland) at a temperature not higher than 45 °C. Hence, to obtain the crude extracts, a wash with distilled water was then carried out. The resuspended extracts and the aqueous extract were lyophilized at −40 °C with a vacuum of 0.035 mBar. Finally, the yield of each extraction was calculated by the Equation (1):

(1) $$Yield\;\left(\%\right)=\left(\frac{Extractedmaterial\left(g\right)}{Initialmaterial\left(g\right)}\right)\times 100$$

### 4.3. Extracts and Drugs Used

For each of the different extracts (methanolic, hexanic and aqueous), four doses were used, one for each experimental group (10, 31.6, 100, and 316 mg/kg; p.o.), which were compared with their respective control group, who only received the solvent consisting of sterile distilled water and a few drops of Tween 80. The administration volume considered in these groups was 1 mL/100 g. In addition, a single dose (10 mg/kg; p.o) of the non-steroidal anti-inflammatory drug diclofenac (AMSA laboratories, Mexico City, Mexico) was used as the reference drug. Finally, 2% formalin (LABESSA Reagents, Mexico City, Mexico) was also used for the induction of the painful stimulus (20 μL; s.c.).

### 4.4. Animals

Briefly, 128 male mice of the NIH strain weighing 25–30 g were used, with food and water available ad libitum under controlled humidity conditions and with a 12 h light/dark cycle. The animals were fasted for 8 h prior to carrying out the experiments. This research project was authorized by Institutional Committee of the National Technological of Mexico Technological Institute of Tuxtla Gutiérrez (protocol number 05-2020/ITTG) and adhered to both the guidelines for experimental research in animals [45] and the Official Mexican Standard (NOM) for the care and use of animals [46].

### 4.5. Evaluation of Antinociceptive Activity

Antinociceptive activity was determined using the formalin model [47], in which previous habituation of 30 min was performed, placing the mouse inside an acrylic chamber (30 cm × 30 cm and 40 cm). After habituation, the administration of the extracts, diclofenac or the vehicle, was carried out. Then, 30 minutes later, the subcutaneous injection of formalin was carried out on the dorsal surface of the right hind limb and the number of paw jerks was recorded in periods of one minute every five minutes for one hour. The response to the stimulus was analyzed in two phases, the first from 1 to 10 min and the second from 15 to 60 min, and the average response of each group was obtained [16].

### 4.6. Toxicity Test

For the determination of the median lethal dose (LD50) the guidelines of the Organization for Economic Cooperation and Development (OECD) were used, using the test known as “up and down”. Five animals were used to evaluate the dose of 2000 mg/kg. Thus, in the evaluation, it must be considered that, if 3–5 mice die, the main test will be carried out to determine the LD_50_ [48].

On the other hand, a biochemical serum analysis was performed, for which all the animals were sacrificed by decapitation after 15 days of observation after the administration of 2000 mg/kg p.o of each of the extracts. Whole blood was centrifuged at 4500 rpm for 15 min to obtain serum. Glucose (mg/dL), cholesterol (mg/dL), triglyceride (mg/dL), albumin (g/dL), total protein (g/dL), uric acid (mg/dL), and amylase (U/L) levels were determined using a Spinlab 14-5331 analyzer (Dieren, The Netherlands).

### 4.7. Identificación de Metabolitos Presentes en el Extracto

#### 4.7.1. Qualitative Analysis by TLC

Thin-layer chromatography (TLC) was performed using silica gel 60 F_254_ plates, 6.6 × 20 cm (0.25 mm thick) Merck^®^ (Mexico City, Mexico), as stationary phase. Then, 30 µL of each extract were applied to 1 cm from the lower limit. The methanolic extract sample was eluted with chloroform-acetone-acetic acid (9:1:0.2), the hexanic extract was eluted with chloroform-petroleum ether-acetic acid -acetone (9:1:0.2:0.5), and the aqueous extract was eluted in chloroform-acetone-ethanol-hexane-toluene (8:1.5:4:0.2:0.6). Chromatography plates were developed as reported by Wagner and Bladt [49].

#### 4.7.2. Quantitative Analysis by Visible Light Spectrophotometry

The following colorimetric methods were used: 2-aminoethyldiphenylborate for the quantification of total flavonoids [50]. The aluminum chloride method was adopted for the quantification of flavones and flavonols [51]. The content of total phenols was assessed using the Folin–Ciocalteu method according to Singleton et al. [52]. The quantification of total saponins was determined according to the report of Wei et al. [53]. The content of total terpenes was quantified according to the description of Fan et al. [54]. Finally, the quantification of alkaloids was performed according to a report by Shamsa et al. [55]. For each of these methods, a Hach Dr 5000 spectrophotometer (Colorado, USA) was used.

#### 4.7.3. Identification of Components in Extracts by Gas Chromatography Coupled to Mass Spectrophotometry

The composition of volatile compounds present in the different extracts was identified using an Agilent Technologies 7890 gas chromatograph coupled to an MSD VL 5975 C mass spectrometry (Wilmington, NC, USA), and the 8270D method was used [56]. The identification of the compounds was carried out by comparing the mass spectra obtained with those of the NIST 2.0 library.

#### 4.7.4. In Silico Analysis of the Most Abundant Compounds

The most abundant secondary metabolites identified in this study was represented in SMILES (simplified molecular-input line-entry system) codes, obtained using the Swiss target software (Swiss Institute of Bioinformatics, version ChEMBL23). All predictions were determined with the same SMILES code. PASS online software (Way2Drug.com, 2011–2022, version 2.0, accessed on 4 July 2022) [37] was used to predict biological activity. This platform calculates the physicochemical and structural properties necessary to perform a comparison with its database. The results are expressed as a probability (*P*) ranging from 0 to 1, where 1 indicates that the event is very likely to occur in vivo and 0 is very unlikely.

Finally, an in-silico toxicity test was performed using the Osiris property explorer software. This software allows determining the properties with high risk of unwanted effects and is shown with a red color, while a green color indicates a behavior in accordance with the evaluated drug or compound [57].

### 4.8. Statistical Analysis

All the data obtained were expressed as the mean ± S.E.M. and statistical significance was determined using ANOVA analysis of variance followed by Tukey’s test for multiple comparisons. The values were considered significantly different at *p* ≤ 0.05, and the statistical program Graphpad Prism version 6.0. was used (GraphPad Software Inc., La Jolla, CA, USA).

## 5. Conclusions

The organic and aqueous extracts of the *Petiveria alliacea* L. leaves are rich in phytochemical compounds, such as terpenes, esters, saponins, alkaloids, anthraquinones, coumarins, anthrones, and flavonoids, some of them with previously reported biological activity. Therefore, it is considered that they could be responsible for the positive antinociceptive effect, which occurred in the neurogenic phase and the inflammatory phase of the formalin model, reducing pain in NIH mice by 35% and 60%, respectively, without showing any difference between the doses evaluated. It should be noted that the extracts were safe and that their use is not considered a health risk, as long as it is not in a prolonged and irresponsible manner.

## Figures and Tables

**Table 1:** Serum biochemical parameters.

Biochemical Indicator	Control Group	Metanoica Extract	Hexanic Extract	Aqueous Extract	Literature Range
Glucose (mg/dL)	127.6 ± 22.61	172.60 ± 31.47 *	113 ± 22.75	170.33 ± 10.02 *	104–290.3
Cholesterol (mg/dL)	130 ± 22.46	107.20 ± 24.34	105.60 ± 21.44	123.33 ± 2.08	67–119.69
Triglyceride (mg/dL)	173.20 ± 41.14	95.20 ± 25.57 **	149.8 ± 41.55	73.00 ± 12.12 ***	54–197.35
Albumin (g/dL)	4.98 ± 0.67	4.60 ± 0.43	4.94 ± 0.28	4.27 ± 0.31	2.1–4.04
Total protein (g/dL)	6.22 ± 0.48	4.66 ± 0.22 ***	6.12 ± 0.28	5.90 ± 0.17	4–6
Uric acid (mg/dL)	7.38 ± 1.05	5.00 ± 2.04 *	6.12 ± 1.06	1.78 ± 0.52 ***	2.06–3.22
Amylase (U/L)	1336 ± 89.23	806.67 ± 233.21 *	1118.5 ± 88.39	1324.40 ± 121.01	607.6–2756

Mean values ± S.E.M., *n* = 5/group. * *p* < 0.05, ** *p* < 0.01, *** *p* < 0.0001 vs. Control group (Saline solution + Tween 80) as determined by one-way ANOVA followed by Tukey test.

**Table 2:** Quantitative analysis of secondary metabolites of the organic and aqueous extracts of *P. alliacea* L.

Secondary Metabolites	Methanolic Extract	Hexanic Extract	Aqueous Extract
Saponins (mg diosgenin/mL)	1.65 ± 0.54	-	0.40 ± 0.02 ***
Total flavonoids (mg rutin eq/mL)	0.52 ± 0.06	0.02 ± 0.00 ***	0.15 ± 0.01 **
Flavones and flavonols (mg quercetin eq/mL)	0.71 ± 0.005	0.008 ± 0.00 ***	0.124 ± 0.03 **
Total Phenols (mg gallic acid/mL)	0.20 ± 0.02	0.029 ± 0.00 **	0.55 ± 0.01 ***
Terpenes (mg ursolic acid/mL)	0.62 ± 0.01	0.55 ± 0.04 *	0.231 ± 0.02 ***
Coumarins (mg umberylferone/mL)	0.142 ± 0.02	0.044 ± 0.01 ***	0.090 ±0.01 *

Mean values ± S.E.M. * *p* < 0.05, ** *p* < 0.01, *** *p* < 0.0001 vs. Methanolic extract as determined by one-way ANOVA followed by Tukey test.

**Table 3:** Metabolites of *P. alliacea* L. leaves identified by GC-MS.

Extract	R.T. (min)	Name	A%	Class
Methanolic	8.812	Ethyl palmitate	4.32	Fatty ester
	9.813	Phytol	48.80	Terpene
	9.974	Ethyl linolenate	17.84	Fatty ester
	13.501	Squalen	7.24	Terpene
Hexanic	5.298	Butylated hydroxytoluene	6.04	Phenolic compound
	9.552	Methyl oleate	12.93	Fatty ester
	11.815	Eicosane	6.25	Hydrocarbon
	13.501	Squalen	10.29	Terpene
Aqueous	8.363	Methyl 14-methylpentadecanoate	7.0	Fatty ester
	9.543	Methyl oleate	14.51	Fatty ester
	9.851	Bis(2-ethylhexyl) maleate	36.27	Ester
	10.195	Octadecyl acetate	1.49	Fatty ester
	12.106	2,4-Bis(1-phenylethyl)phenol	3.03	Phenolic compound

R.T (retention time); A% (percent abundance).

**Table 4:** PASSonline activities.

Extract	Compounds	Antinociceptive		Antiinflammatory	
		Pa	Pi	Pa	Pi
Methanolic	Ethyl palmitate	0.472	0.054	0.600	0.032
Methanolic	Phytol	0.300	0.182	0.458	0.070
Methanolic	Ethyl linolenate	0.509	0.031	0.827	0.005
Methanolic/ Hexanic	Squalene	0.474	0.053	0.701	0.016
Hexanic	Butylated hidroxytoluene	0.498	0.037	0.803	0.006
Hexanic Aqueous	Methyl oleate	0.573	0.011	0.607	0.030
Hexanic	Eicosane	0.595	0.012	0.424	0.004
Aqueous	Methyl 14-methylpentadecanoate	0.490	0.042	0.392	0.1
Aqueous	Bis(2-ethylhexyl) maleate	0.331	0.160	0.605	0.030
Aqueous	Octadecyl acetate	0.455	0.067	0.717	0.014
Aqueous	2,4-bis(1-phenylethyl) phenol	0.555	0.014	0.318	0.145

Pa = probability to be active; Pi = probability to be inactive.

**Table 5:** Toxicity Risks by OSIRIS Property Explorer.

Extract	Compounds	M.	T.	I.	R.E.
Methanolic	Ethyl palmitate	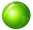	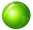	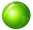	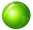
Methanolic	Phytol	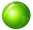	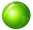	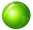	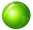
Methanolic	Ethyl linolenate	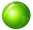	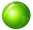	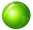	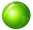
Methanolic/Hexanic	Squalene	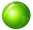	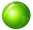	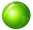	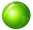
Hexanic	Butylated hidroxytoluene	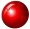	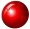	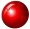	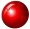
Hexanic Aqueous	Methyl oleate	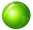	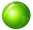	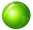	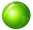
Hexanic	Eicosane	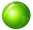	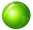	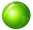	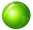
Aqueous	Methyl 14-methylpentadecanoate	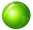	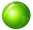	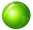	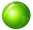
Aqueous	Bis(2-ethylhexyl) maleate	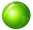	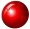	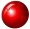	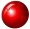
Aqueous	Octadecyl acetate	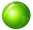	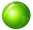	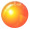	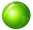
Aqueous	2,4-bis(1-phenylethyl) phenol	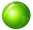	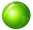	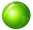	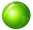

M: Mutagenic, T: Tumorigenic, I: Irritability, R.E: Reproductive effect.

## Data Availability

The data are contained within the article.
